# Use of Antidepressants Decreased After Initiation of ADHD Treatment in Adults—A Finnish Nationwide Register Study Describing Use of ADHD and Non‐ADHD Medication in People With and Without ADHD

**DOI:** 10.1111/acps.70007

**Published:** 2025-06-27

**Authors:** Elisa Westman, Tuire Prami, Alvar Kallio, Ilona Iso‐Mustajärvi, Joel Jukka, Paavo Raittinen, Maarit J. Korhonen, Anita Puustjärvi, Sami Leppämäki

**Affiliations:** ^1^ Takeda Oy Helsinki Finland; ^2^ University of Helsinki Helsinki Finland; ^3^ Oriola Espoo Finland; ^4^ Kuopio University Hospital Kuopio Finland; ^5^ Terveystalo Helsinki Finland

**Keywords:** antidepressants, attention‐deficit hyperactivity disorder, drug holidays, medication pathways, persistence, primary adherence

## Abstract

**Introduction:**

ADHD is often associated with comorbid psychiatric conditions. Differential diagnosis between other conditions and ADHD is not always clear, and patients are sometimes initially treated for another disorder instead. ADHD diagnosis and appropriate ADHD treatment potentially reduce the need for medication of the other disorder. Reaching high adherence to and persistence with ADHD medication is challenging. This nationwide cohort study aimed to describe not only ADHD medication use but also the use of other drugs in ADHD patients compared to controls.

**Methods:**

Nationwide care and prescription registers were used to identify incident ADHD patients of any age between 2015 and 2020. Four controls were matched to each ADHD patient by age, gender, and residence. Analyses included data from 1.1.2010 to 31.12.2021.

**Results:**

Study cohort included 66,146 ADHD patients and 256,270 controls, with a total follow‐up of 1,123,412 years. Sustained and extended‐release methylphenidate were the two most commonly used first‐line ADHD drugs across all age groups. Simultaneous use of different ADHD drugs was rare. Primary adherence was very high, with 95% of the patients purchasing their prescribed medication in general and 80% doing so within 10 days. Persistence with medication was the highest among the youngest patients. A decrease in purchases was observed during the summer holidays in school‐age children and adolescents. In adults, antidepressant use often preceded ADHD diagnosis and decreased after ADHD treatment initiation, unlike in controls at the same time. In young children, antibiotics and anti‐inflammatory drug use was higher in ADHD patients than in controls, especially before ADHD identification.

**Conclusion:**

As far as we know, this is the first study to describe changes in the use of non‐ADHD medications in relation to ADHD identification. In adults, antidepressant use decreased after ADHD treatment initiation, and in children, antibiotic and anti‐inflammatory use showed more prominent decrease compared to controls of the same age. The data indicated high primary adherence to ADHD medication, and the youngest children remained on continuous ADHD medication the longest. The effect of summer holidays was visible in the purchase data.

## Graphical abstract

Use of ADHD and non‐ADHD medication in people with and without ADHD in Finland.

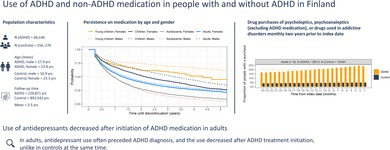

**Summary**

- Significant outcomes
  ○ Antidepressant use was common in adults prior to identification of ADHD but decreased substantially after initiation of treatment for ADHD.
  ○ Children who were later diagnosed with ADHD used more antibiotics and anti‐inflammatory drugs than matched controls. After the initiation of treatment for ADHD, the use of these drugs decreased more than in controls.
  ○ Primary treatment adherence in all age groups was very high, as most patients purchased the ADHD medication prescribed for them. Persistence with ADHD medication was the highest in children and the lowest in adolescents.
- Limitations
  ○ This study included data obtained from the Finnish national registers, which had limitations related to the coverage of private healthcare data.
  ○ Data on adherence and persistence were based on drug purchases from community pharmacies, and analyses on continuous medication assumed that the medication had been used as indicated. However, especially in the case of stimulants, we cannot rule out the possibility of misuse and dealing.

## 1 Introduction

Attention‐deficit hyperactivity disorder (ADHD) symptoms can often be managed by combining pharmacological and non‐pharmacological treatments. Medication options available in Europe include psychostimulants and non‐psychostimulants [1, 2], both of which reduce core symptoms and negative outcomes related to ADHD [3, 4]. For children and adolescents, the recommended first‐line treatment is methylphenidate [1, 5, 6, 7]. For adults, first‐line drug treatment can be either a psychostimulant (lisdexamfetamine or methylphenidate), or a non‐stimulant, atomoxetine [5]. In the previous version of the Finnish Current Care Guidelines, methylphenidate was recommended as the first‐line drug treatment also for adults [8]. In line with the guidelines [1, 8], recent register studies from Finland and Sweden have shown that the most common ADHD medication has been methylphenidate, used by about 70% of ADHD patients of any age receiving medication in these two Nordic countries [9, 10].

Achieving high adherence to and persistence with ADHD medication has been shown to be challenging from patients', their families', and clinicians' point of view [11]. According to a systematic review, treatment duration averaged 5.6 months, and only 23% of children and adolescents were adherent to treatment at 12 month [12]. According to a recent Danish nationwide registry study, 24% of men and 29% of women were estimated to persist with medication at least for 5 years [13]. Switching ADHD medication two to three times or more decreased the risk of discontinuation of any ADHD medication significantly during the 5‐year follow‐up. A large population‐based database study from nine countries revealed that the ADHD treatment discontinuation rate 1 to 5 years after the initiation was the lowest in children and the highest in young adults and adolescents, being the very highest in patients 18–19 years of age [14]. No differences in persistence between sexes were seen in this study.

ADHD is known to be linked to comorbid psychiatric conditions, such as mood disorders, anxiety disorders, or depression, and recognition of ADHD can be challenging due to these overlapping symptoms [3, 15, 16]. In adults, comorbid psychiatric disorders with ADHD include substance use disorder, bipolar disorder, anxiety, and depression [17]. Of somatic conditions, obesity, sleep disorders, and asthma are well‐documented comorbidities in adult ADHD patients [18]. A meta‐analysis of childhood physical health and ADHD showed a significant association of ADHD with childhood injuries, infections, sleep problems, and allergies [19]. Recently, it was shown that sensory and neurological conditions in childhood were associated with higher ADHD symptoms at age 17 [20]. The use of medications—both psychiatric and non‐psychiatric—could be used as a proxy for comorbid diseases and disorders. To what extent getting a diagnosis of ADHD or initiating drug treatment for ADHD changes the use of other medications has—at least to our knowledge—not been studied before.

The aim of the present study was to describe ADHD patients' ADHD medication use by age and gender. In addition, medication patterns of other drugs used by ADHD patients compared to controls were studied in a nationwide Finnish cohort.

## 2 Materials & Methods

This was a nationwide cohort study utilizing secondary data from national databases in Finland. Nationwide care registers, as well as nationwide prescription registers, including prescriptions, dispensations, reimbursed purchases, and entitlements to higher than regular medication reimbursement were used to identify people with ADHD. In addition to diagnoses and medication, sociodemographics and dates of death were obtained from different administrative registers. Personal‐level data were linked across data sources by unique personal identity numbers, which were pseudonymized by the authorities. A more detailed description of the cohort identification can be found in a previous publication by the same research group [10].

The study population consisted of incident ADHD patients who fulfilled any of the ADHD identification criteria (prescription or drug purchase of dexamphetamine, methylphenidate, atomoxetine, lisdexamphetamine, or guanfacine, reimbursement status, or diagnosis for ADHD) for the first time during 2015–2020, and none of the criteria during the history period 2010–2014. The index date for each ADHD patient was the first date when any of the identification criteria were met. Follow‐up started at index date and ended at moving abroad, death, or 31‐12‐2021, whichever occurred first.

Each ADHD patient was matched with four controls based on year of birth (+/− 1 year), gender, and place of residence in the beginning of 2015 by authorities. The ADHD population and control population did not overlap; thus, the controls were people without any identification criteria for ADHD. For some people, no controls or only a limited number of controls were identified. In analyses where the ADHD population was compared to the control population, only ADHD patients with at least one control were considered. Controls were given the same index date as the respective ADHD patient.

ADHD and other medications were identified from the prescription registers using Anatomical Therapeutic Chemical (ATC) codes. For methylphenidate, Nordic product numbers were further used to classify products according to their drug‐releasing profile: instant‐release, sustained‐release, or extended‐release methylphenidate. Primary adherence to ADHD medication was analyzed based on the time from the first ADHD drug prescription to the date of the first ADHD drug purchase. Persistence with ADHD medication was defined as the time from the first to the last purchase plus 90 days to use the supply. If there were no new purchases in 183 days after the previous purchase, the medication was considered discontinued. The Kaplan–Meier estimator was used to describe primary adherence to and persistence with ADHD medication. Concomitant ADHD medication use was defined as purchasing two different drugs in alternating order, both for at least twice within 90 days. ADHD medication pathways were defined as the purchase of a new drug any time after the purchase of the previous drug.

To analyze prespecified ATC class N (*nervous system*) medication prior to the index date in both ADHD and control populations, the proportion of people with N05* (*psycholeptics*), N06* (*psychoanaleptics*), and N07B* (*drugs used in addictive disorders*) drug purchases in each age group was calculated monthly for 2 years (This analysis excluded ADHD medications N06BA02, N06BA04, N06BA09, and N06BA12, which by definition could not be used by controls or not even by ADHD people prior to index date.).

The Kaplan–Meier estimator was used for the 20 most purchased drugs (five‐digit ATC code subgroups) to estimate the proportion of people with or without ADHD who used the medication within a specific time window. The absolute change (%‐point) was presented for 12–24 months after the index date as compared to 0–12 months prior to the index date, and 0–12 months after the index date was considered as a respite for the change to occur.

The study permit was issued by the Finnish Social and Health Data Permit Authority Findata (Dnro THL/3461/14.02.00/2022). Data were stored and analyzed in a validated secure data environment provided by Statistics Finland. Analyses were performed using R version 4.2.2. Results were quality‐checked by dual programming. Results with frequencies of less than five individuals were not reported to ensure the privacy of individuals.

## 3 Results

### 3.1 Study Cohort

Altogether, 66,146 incident ADHD patients and 256,270 controls were included in the analyses. At least one matched control was identified for 64,752 ADHD patients, and 96.0% of them (62,147 ADHD patients) had four controls. The mean age at the index date was 17.0 (median 11) years for males with ADHD and 23.4 (median 21) for females with ADHD, and 16.9 (median 11) and 23.3 (median 21) for the matched male and female controls, respectively. The follow‐up time was 229,871 years for ADHD patients and 893,542 years for controls. The mean follow‐up time was 3.5 years for both the ADHD patients and the controls.

### 3.2 Non‐ADHD Medication Use Before ADHD Identification With Comparison to Matched Non‐ADHD Controls

**FIGURE 1:**
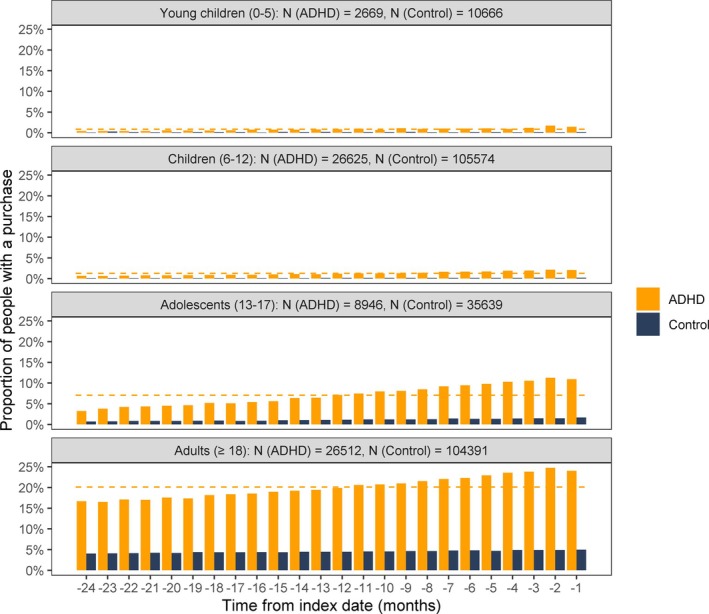
Proportion of ADHD patients and their controls with a drug purchase of psycholeptics, psychoanaleptics (excluding ADHD medication), or drugs used in addictive disorders monthly 2 years prior to the index date in different age categories. The dashed line indicates the mean over time in the ADHD group.

Drug purchases of *psycholeptics*, *psychoanaleptics* (excluding ADHD drugs), or *drugs used in addictive disorders* (i.e., in nicotine, alcohol, and opioid dependence) within 2 years prior to the index date are shown in Figure 1. These purchases were the most common in adults, and in adult and adolescent ADHD patients, there was a remarkable difference in purchases of these medications compared to controls. In all ADHD patients, there was a slight increase in purchases of these drugs when getting closer to the index date.

The number of different *psycholeptics*, *psychoanaleptics* (excluding ADHD drugs), and *drugs used in addictive disorders* purchased up to 2 years before the index date is presented in Table 1. More than 90% of the children with ADHD did not receive any of these medications prior to the index date. About one‐third of the adults with ADHD did not receive this type of medication, but another third received three or more different drugs. In adults, there was a discernible difference, but in adolescents, a notable difference in the use of these drugs between genders, as it was more common in females than in males both among ADHD patients and controls.

**TABLE 1:** Number and proportion of ADHD patients and controls according to the number of distinct psycholeptics, psychoanaleptics (excluding ADHD medication), and drugs used in addictive disorders purchased up to 2 years prior to the index date by gender and age at index. (The age category 0–5 years is excluded from this table to avoid presenting results *n* < 5.).

	Females		Males	
	ADHD, *N* (%)	Control, *N* (%)	ADHD, *N* (%)	Control, *N* (%)
Children (6–12 years)				
0 drugs	5179 (91.8)	22,012 (98.3)	19,481 (92.8)	81,663 (98.2)
1 drug	326 (5.8)	350 (1.6)	1190 (5.7)	1343 (1.6)
2 drugs	98 (1.7)	27 (0.1)	225 (1.1)	124 (0.1)
3 or more drugs	37 (0.7)	7 (0.0)	89 (0.4)	48 (0.1)
Adolescents (13–17 years)				
0 drugs	2418 (60.1)	14,662 (91.6)	3832 (77.9)	19,005 (96.8)
1 drug	635 (15.8)	667 (4.2)	560 (11.4)	415 (2.1)
2 drugs	411 (10.2)	321 (2.0)	256 (5.2)	136 (0.7)
3 or more drugs	562 (14.0)	353 (2.2)	272 (5.5)	80 (0.4)
Adults (≥ 18 years)				
0 drugs	4367 (32.0)	41,631 (77.6)	5170 (40.1)	43,371 (85.5)
1 drug	2757 (20.2)	6018 (11.2)	2425 (18.8)	3790 (7.5)
2 drugs	2146 (15.7)	2726 (5.1)	1707 (13.3)	1666 (3.3)
3 or more drugs	4363 (32.0)	3285 (6.1)	3577 (27.8)	1904 (3.8)

The 20 most purchased drugs by the ADHD patients prior to the index date are presented in Table 2. The use of *selective serotonin reuptake inhibitors*, *benzodiazepine derivatives*, *diazepines, oxazepines, thiazepines and oxepines*, and *other antidepressants* was more common before the index date in adult ADHD patients but decreased during the second follow‐up year, while the use of these drugs increased in controls. The same phenomenon was seen in the youngest children with systemic and topical antibiotics, selective beta‐AR‐agonists, glucocorticoids, and certain antihistamines; the use of these drugs was more common in 0–5‐year‐old ADHD patients compared to their controls before the index date, but it also decreased more during the follow‐up in ADHD children compared to controls. The use of *other antiepileptics* was four times more common in adult ADHD patients than in controls.

**TABLE 2:** Percentage (%) of ADHD patients and controls divided by age groups using the 20 most purchased drugs (the 4th level ATC code subgroup) of ADHD patients 0–12 months prior to the index date, and absolute change (%‐point) in proportion compared to the initial proportion 12–24 months after the index date by Kaplan–Meier estimate. (Months 0–12 after the index date were considered as a respite for the change to occur.).

Chemical, pharmacological or therapeutic subgroup	All age groups		Young children (0–5 years)		Children (6–12 years)		Adolescents (13–17 years)		Adults (≥ 18 years)	
	ADHD	Control	ADHD	Control	ADHD	Control	ADHD	Control	ADHD	Control
Propionic acid derivatives (M01AE)	19.3 (−1.0)	14.1 (−0.4)	17.5 (−5.3)	12.8 (−3.0)	9.6 (−1.6)	8.5 (−1.6)	14.7 (1.6)	10.1 (1.6)	30.8 (−0.4)	21.4 (0.8)
Other antidepressants (N06AX)	12.6 (−0.7)	2.0 (0.6)	0.0 (0.0)	0.0 (0.0)	< 5 (−)^a^	0.0 (0.0)	4.5 (3.9)	0.5 (1.2)	29.4 (−2.7)	4.6 (1.3)
Selective serotonin reuptake inhibitors (N06AB)	11.4 (−1.4)	2.7 (0.6)	< 5 (−)^a^	0.0 (−)	1.0 (1.3)	0.1 (0.2)	15.2 (1.9)	2.4 (2.3)	21.7 (−5.2)	5.7 (0.5)
Penicillins with extended spectrum (J01CA)	11.1 (−4.1)	9.2 (−3.1)	23.5 (−11.9)	18.6 (−8.6)	11.6 (−6.2)	10.4 (−5.2)	8.5 (−1.6)	6.0 (−0.7)	10.3 (−1.9)	8.0 (−1.2)
Selective beta‐2‐adrenoreceptor agonists (R03AC)	10.9 (−1.0)	7.4 (−0.7)	13.9 (−2.7)	9.5 (−1.6)	9.7 (−1.8)	8.0 (−1.1)	9.8 (−1.9)	6.7 (−1.1)	12.2 (0.4)	6.8 (−0.0)
Anilides (N02BE)	10.7 (0.2)	7.2 (0.2)	9.2 (−2.5)	7.0 (−2.3)	5.2 (−1.0)	4.5 (−0.7)	8.0 (1.2)	4.7 (1.1)	17.3 (1.6)	10.8 (1.3)
Corticosteroids (R01AD)	9.3 (−0.4)	7.2 (0.3)	2.8 (1.9)	2.6 (1.9)	5.8 (0.1)	5.6 (0.7)	8.7 (−1.1)	7.2 (−0.4)	13.6 (−0.6)	9.3 (0.1)
First‐generation cephalosporins (J01DB)	9.1 (−1.7)	7.0 (−1.2)	9.0 (−2.3)	7.8 (−1.4)	7.5 (−2.2)	6.7 (−1.9)	8.3 (−1.0)	5.5 (0.1)	11.0 (−1.3)	7.8 (−0.8)
Benzodiazepine derivatives (N05BA)	7.5 (−0.5)	1.5 (0.1)	1.4 (−0.8)	0.3 (−0.2)	0.4 (−0.1)	0.1 (−0.0)	1.2 (0.7)	0.3 (0.3)	17.3 (−0.9)	3.3 (0.4)
Diazepines, oxazepines, thiazepines and oxepines (N05AH)	7.1 (0.3)	1.3 (0.3)	< 5 (−)^a^	0.0 (−)	0.3 (0.8)	0.0 (0.1)	8.7 (2.4)	1.1 (0.8)	14.1 (−0.6)	2.8 (0.4)
Other antihistamines for systemic use (R06AX)	7.0 (−0.2)	5.7 (0.1)	7.8 (−0.9)	6.8 (0.2)	6.3 (−0.8)	6.2 (−0.3)	5.8 (0.6)	4.9 (0.1)	7.9 (0.2)	5.3 (0.4)
Proton pump inhibitors (A02BC)	6.4 (0.4)	3.5 (0.4)	1.6 (−0.3)	0.8 (0.2)	1.1 (−0.1)	0.8 (0.1)	2.5 (0.7)	1.3 (0.4)	13.4 (1.1)	7.2 (0.8)
Antibiotics (S01AA)	6.1 (−1.7)	5.5 (−1.4)	15.0 (−7.0)	14.0 (−6.0)	7.2 (−2.7)	6.6 (−2.6)	4.6 (−1.3)	3.5 (−0.1)	4.7 (−0.2)	4.3 (−0.1)
Glucocorticoids (R03BA)	5.7 (−0.9)	3.9 (−0.4)	7.9 (−1.4)	4.8 (−0.4)	6.1 (−1.5)	4.8 (−0.8)	4.7 (−1.4)	3.3 (−0.7)	5.5 (−0.1)	3.2 (−0.0)
Melatonin receptor agonists (N05CH)	5.6 (5.2)	1.1 (0.5)	1.2 (7.3)	0.1 (0.0)	1.2 (6.7)	0.1 (0.2)	9.3 (5.6)	1.3 (1.0)	9.3 (3.3)	2.0 (0.8)
Beta‐lactamase sensitive penicillins (J01CE)	5.6 (−1.1)	4.3 (−0.7)	3.7 (−0.1)	2.9 (0.3)	4.2 (−1.1)	3.6 (−0.8)	5.9 (0.2)	3.7 (0.5)	7.2 (−1.6)	5.4 (−0.9)
Piperazine derivatives (R06AE)	5.0 (0.5)	3.9 (0.1)	3.3 (0.6)	2.8 (0.2)	4.2 (0.7)	3.8 (0.4)	5.4 (0.3)	4.3 (−0.2)	5.7 (0.4)	3.9 (−0.1)
Other centrally acting agents (M03BX)	4.0 (−0.1)	1.9 (0.0)	< 5 (−)^a^	< 5 (−)^a^	< 5 (−)^a^	0.0 (0.0)	0.5 (0.3)	0.2 (0.3)	9.6 (−0.1)	4.6 (0.1)
Sympathomimetics (R01BA)	3.9 (−0.7)	3.0 (−0.1)	0.0 (−)	0.0 (−)	0.2 (0.2)	0.2 (0.3)	2.9 (−0.3)	2.5 (0.2)	8.4 (−1.7)	6.4 (−0.5)
Other antiepileptics (N03AX)	3.9 (0.7)	0.9 (0.2)	0.8 (0.4)	0.1 (0.0)	0.4 (0.2)	0.1 (0.0)	1.0 (0.6)	0.4 (0.3)	8.7 (1.4)	2.0 (0.3)

^a^ Results with frequencies < 5 individuals were not reported.

### 3.3 ADHD Medication Use in the Incident ADHD Cohort

Of incident ADHD patients, 83.1% purchased some ADHD medication during follow‐up (Table 3). The most purchased product was extended‐release methylphenidate by 56.5% of these patients. ADHD medication use was more common in boys than in girls in the age groups of 0–5‐year‐old and 6–12‐year‐old children, whereas in adolescents and adults, it was more common in females than in males.

**TABLE 3:** Number of patients purchasing ADHD medication at least once during follow‐up with percentages from ADHD population by gender and age at the index date.

	Total, *N*	Any medication, *N* (%)	MPH (E), *N* (%)	MPH (S), *N* (%)	MPH (I), *N* (%)	LDX, *N* (%)	ATX, *N* (%)	DX, *N* (%)	GUA, *N* (%)
Young children (0–5 years)									
Female	544	369 (67.8)	173 (31.8)	333 (61.2)	57 (10.5)	63 (11.6)	61 (11.2)	< 5 (−)^a^	12 (2.2)
Male	2175	1661 (76.4)	911 (41.9)	1503 (69.1)	402 (18.5)	401 (18.4)	308 (14.2)	25 (1.1)	102 (4.7)
Total	2719	2030 (74.7)	1084 (39.9)	1836 (67.5)	459 (16.9)	464 (17.1)	369 (13.6)	25 (0.9)	114 (4.2)
Children (6–12 years)									
Female	5736	4784 (83.4)	2695 (47.0)	3732 (65.1)	736 (12.8)	622 (10.8)	483 (8.4)	35 (0.6)	98 (1.7)
Male	21,455	18,580 (86.6)	10,606 (49.4)	15,118 (70.5)	3308 (15.4)	2878 (13.4)	2061 (9.6)	148 (0.7)	628 (2.9)
Total	27,191	23,364 (85.9)	13,301 (48.9)	18,850 (69.3)	4044 (14.9)	3500 (12.9)	2544 (9.4)	183 (0.7)	726 (2.7)
Adolescents (13–17 years)									
Female	4075	3483 (85.5)	2758 (67.7)	1554 (38.1)	316 (7.8)	468 (11.5)	481 (11.8)	20 (0.5)	37 (0.9)
Male	5011	4186 (83.5)	3135 (62.6)	1733 (34.6)	359 (7.2)	419 (8.4)	551 (11.0)	29 (0.6)	70 (1.4)
Total	9086	7669 (84.4)	5893 (64.9)	3287 (36.2)	675 (7.4)	887 (9.8)	1032 (11.4)	49 (0.5)	107 (1.2)
Adults (≥ 18 years)									
Female	13,942	11,406 (81.8)	9010 (64.6)	4728 (33.9)	1839 (13.2)	2801 (20.1)	1486 (10.7)	528 (3.8)	40 (0.3)
Male	13,208	10,493 (79.4)	8100 (61.3)	3662 (27.7)	1619 (12.3)	2542 (19.2)	1428 (10.8)	508 (3.8)	51 (0.4)
Total	27,150	21,899 (80.7)	17,110 (63.0)	8390 (30.9)	3458 (12.7)	5343 (19.7)	2914 (10.7)	1036 (3.8)	91 (0.3)
Total female	24,297	20,042 (82.5)	14,636 (60.2)	10,347 (42.6)	2948 (12.1)	3954 (16.3)	2511 (10.3)	583 (2.4)	187 (0.8)
Total male	41,849	34,920 (83.4)	22,752 (54.4)	22,016 (52.6)	5688 (13.6)	6240 (14.9)	4348 (10.4)	710 (1.7)	851 (2.0)
Total	66,146	54,962 (83.1)	37,388 (56.5)	32,363 (48.9)	8636 (13.1)	10,194 (15.4)	6859 (10.4)	1293 (2.0)	1038 (1.6)

Abbreviations: ATX, atomoxetine; DX, dexamphetamine; GUA guanfacine; LDX, lisdexamphetamine; MPH (E), methylphenidate (extended‐release); MPH (I), methylphenidate (instant release); MPH (S), methylphenidate (sustained‐release).

^a^ Results with frequencies < 5 individuals were not reported.

When observing primary adherence, more than 80% of the ADHD patients purchased the prescribed medication within 10 days of the prescription, and less than 5% did not purchase the prescribed medication during the follow‐up (Figure S1).

**FIGURE 2:**
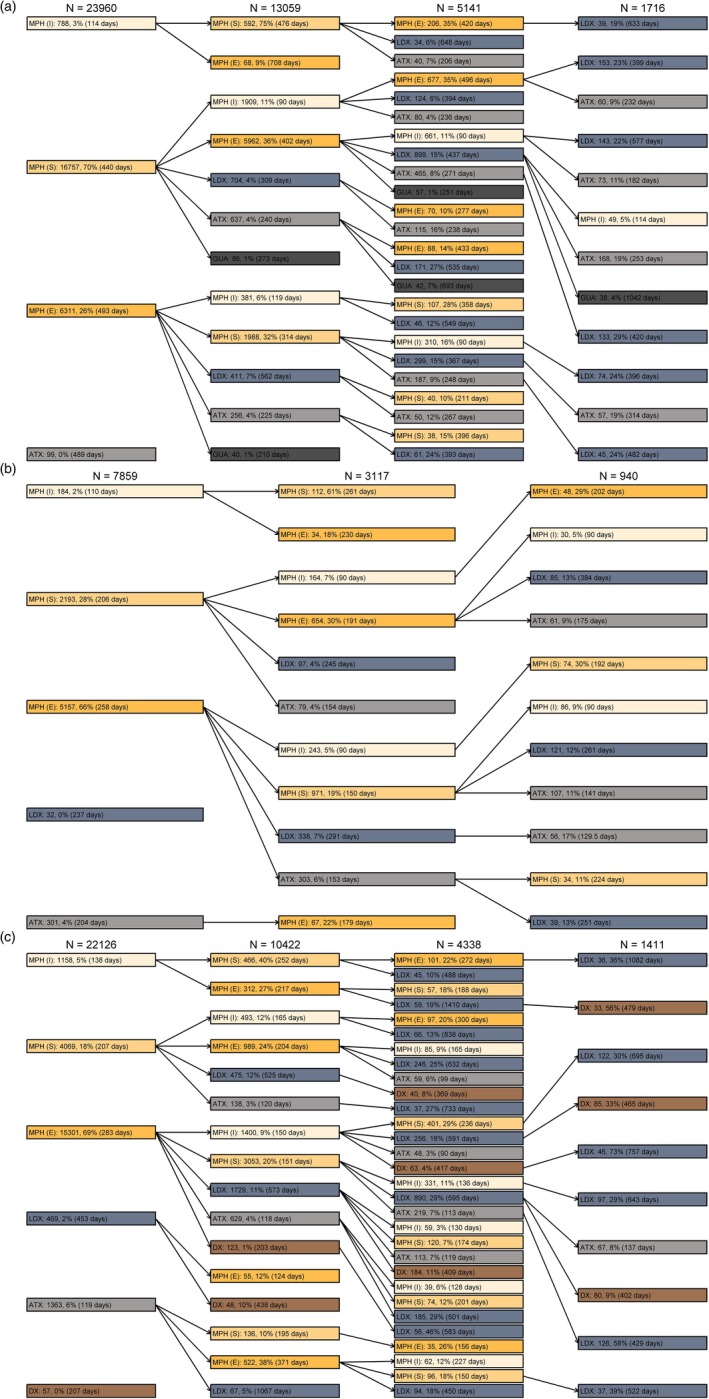
Medication pathways in (a) 6–12 years, (b) 13–17 years, and (c) in ≥ 18 years old ADHD patients indicating the first purchase of each drug: ATX, atomoxetine; DX, dexamphetamine; GUA guanfacine; LDX, lisdexamphetamine; MPH (I), methylphenidate (instant release); MPH (S), methylphenidate (sustained‐release), MPH (E), methylphenidate (extended‐release). Only nodes with an absolute frequency of at least 30 are included. Days refer to the median time from initiation to discontinuation of the current drug estimated using Kaplan–Meier estimator (The age category 0–5 years is excluded from this figure to avoid presenting results *n* < 5.).

Figure 2 shows medication pathways as the order of different ADHD drugs by age groups. In 6–12‐year‐old children, sustained‐release methylphenidate was the most used first ADHD medication; 70% of ADHD patients in this age groups used it as the first medication. In adolescents and adults, the first product was extended‐release methylphenidate (in 66% and in 69%, respectively). Children used the first sustained or extended‐release methylphenidate product for a median of over 400 days before receiving another ADHD medication; in adolescents and adults, the median was over 200 days. In every age group, the most common drugs of choice for the first and second product were sustained and extended‐release methylphenidate, in either order.

Concomitant use of two or more ADHD drugs was uncommon. In 6–12‐year‐old children and in 13–17‐year‐old adolescents, the most common combination was instant‐release and sustained‐release methylphenidate. In adults, it was instant‐release and extended‐release methylphenidate.

**FIGURE 3:**
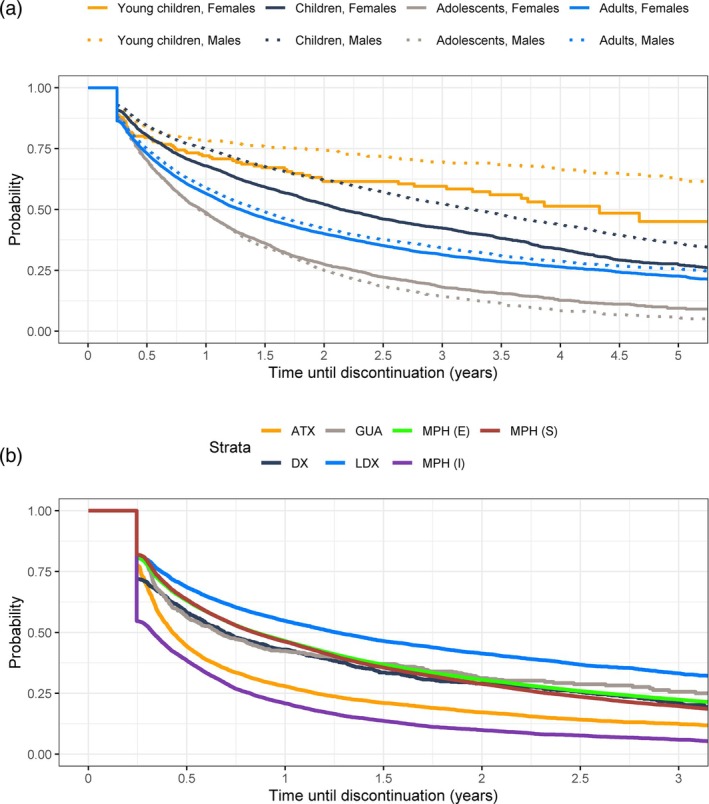
Kaplan–Meier estimators for the time until discontinuation of (a) any ADHD medication by age groups (based on the age at the time of the first purchase) and (b) separate ADHD drugs [ATX, atomoxetine; DX, dexamphetamine; GUA guanfacine; LDX, lisdexamphetamine; MPH (I), methylphenidate (instant release); MPH (S), methylphenidate (sustained‐release), MPH (E), methylphenidate (extended‐release)].

Persistence of ADHD medication is presented in Figure 3 for different age groups and genders (a) and for separate ADHD drugs (b). Persistence was the lowest in the adolescents (Figure 3a). In this age group, less than 50% of patients who initiated medication were on medication 1 year after the first purchase. The youngest children persisted the longest. In 0–5‐ and 6–12‐year‐old children, boys stayed on the medication longer than girls. Of separate ADHD drugs, persistence was the highest with lisdexamphetamine and the lowest with atomoxetine and instant‐release methylphenidate (Figure 3b). Almost half of the patients who initiated instant‐release methylphenidate withdrew this medication within 3 months.

**FIGURE 4:**
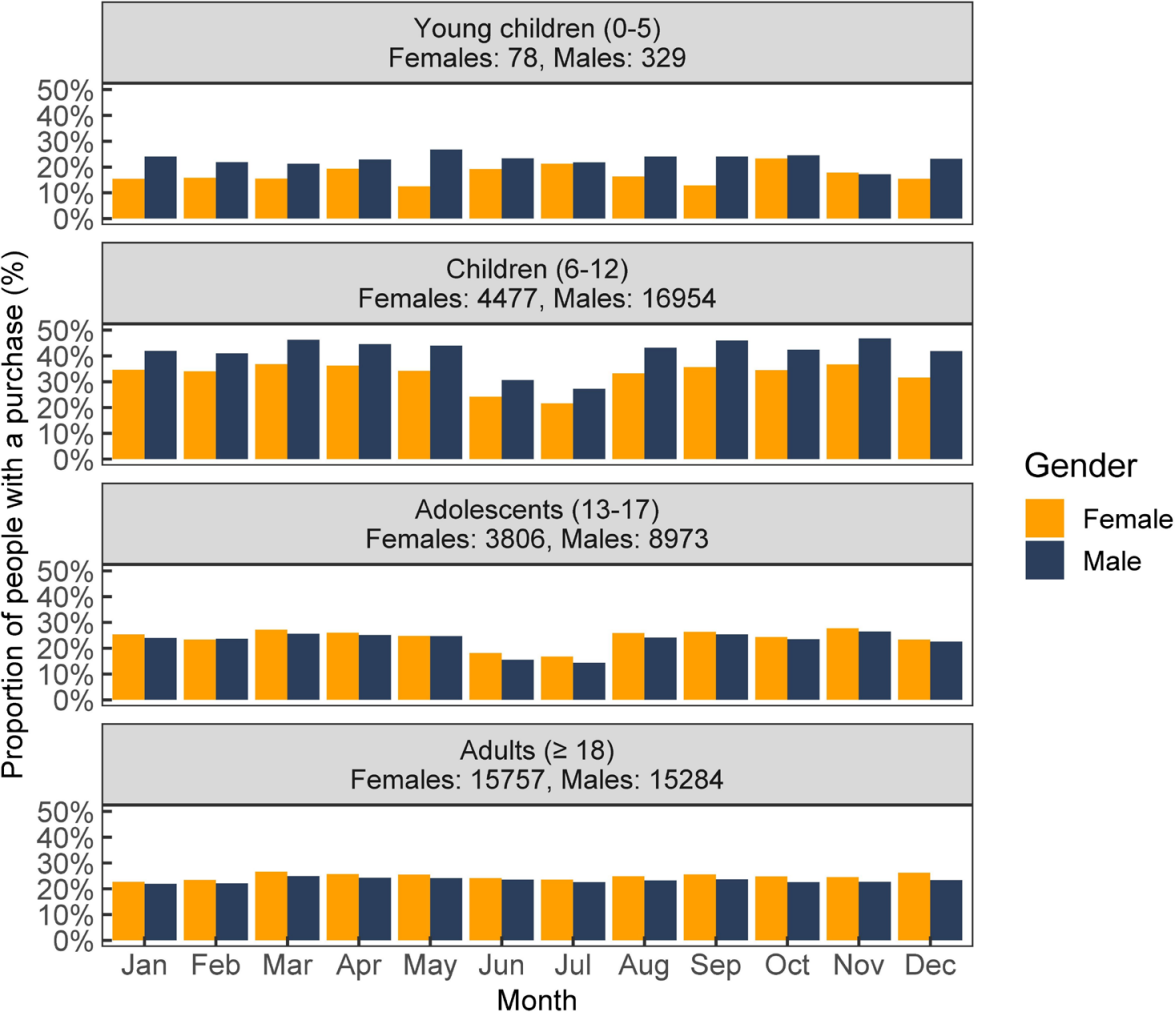
ADHD medication purchasing behavior by calendar months in 2021 divided by gender and age groups.

To assess possible drug holidays, purchasing behavior by calendar months in 2021 is presented in Figure 4. Fewer purchases of ADHD drugs were seen in 6–12‐year‐old children and 13–17‐year‐old adolescents in June and July (school holiday months in Finland) compared to other calendar months.

## 4 Discussion

This nationwide cohort study highlights that ADHD identification in adulthood is often preceded by antidepressant use. As far as we know, this is the first study reporting changes in medication use before and after ADHD identification. Antidepressant use in adult ADHD patients decreased after ADHD treatment initiation, contrasting with the increase observed in their matched controls. The older the patients were at the time of ADHD diagnosis, the more they had used psycholeptics, psychoanaleptics, and drugs for dependencies prior to it, and in every age group, these medications use was remarkably more common in ADHD patients than in their respective controls. Notably, females were more likely than males to use these medications prior to diagnosis, possibly due to a misinterpretation of ADHD symptoms as anxiety or depression. On the other hand, anxiety and depression may be a consequence of untreated ADHD, but just as well it may be independent from ADHD since the use of these drugs was more common also in control young and adult women than in control men of the same age.

Previous literature in relation to ADHD and changes in antidepressant use is scarce. Discontinuation of antidepressant medication has been discussed to be a concern in depression management in people diagnosed with ADHD [21, 22], and it has been associated with low antidepressant effectiveness among patients with ADHD [23]. We would like to rather interpret that the adjustment of pharmacotherapy before and after initiating ADHD treatment is a dynamic process, and previous medications may indicate other symptoms provoked by ADHD, which may have delayed the diagnosis of ADHD itself. On the other hand, it is known that ADHD is often comorbid with other psychiatric disorders, and alcohol and substance abuse [24]. Thus, especially in adults, the diagnostic assessment should both include a review of past and present symptoms and functional performance, as well as assessment of a broader spectrum of psychiatric and somatic conditions.

ADHD is a frequent comorbidity of epilepsy patients of all ages [25]. In our data, the use of certain antiepileptics was four times more common in adult ADHD patients than in controls, and this difference is likely to be driven by the use of these drugs as mood stabilizers. Pregabalin and gabapentin, also used for pain management, have misuse potential and require careful prescribing, especially for those with substance abuse history. Lamotrigine use may reflect comorbid epilepsy, treatment‐resistant depression, or bipolar disorder commonly seen in ADHD patients. In addition, some of the antipsychotics and selective serotonin reuptake inhibitors that appeared in our list of 20 most common comedication, may be used for the treatment of bipolar disorder too. On the other hand, we cannot clearly draw a conclusion about a direct link between bipolar disorder and ADHD based on our own data. In some cases, it may involve a possible misdiagnosis of ADHD in patients with bipolar disorder or the use of ADHD medication as supportive treatment for bipolar disorder.

Use of *melatonin receptor agonists* was more than four times as common and use of *proton pump inhibitors* almost twice as common in ADHD patients of all age groups compared to controls already before the index date, and the use of melatonin further increased after ADHD treatment initiation. Both might be linked to psychological effects and provocative addition of insomnia and stomach pain. In every case, both drugs can be purchased with and without prescription, and melatonin even outside pharmacies; thus, our data most probably do not show all purchases. The ADHD population, however, is in intense contact with health care even before ADHD diagnosis, thus they may receive the prescription more likely than their respective controls.

In the youngest children, the use of systemic and topical antibiotics, selective beta‐AR‐agonists, glucocorticoids, and certain antihistamines was more common in ADHD patients than in their controls. In addition, the use of these drugs was more common before ADHD identification than after that, and the decrease by time and aging was more prominent in ADHD patients than in controls. Earlier, the association between ADHD and exposure to antibiotics has widely been studied from the developmental point of view, based on which the results at the population level are controversial [26, 27, 28]. On the other hand, association between early life antibiotic exposure and ADHD may be partially explained by genetic and familial factors [29]. In addition to bacterial infections, ADHD has been linked to bronchial asthma [30] and atopic dermatitis [31]. In our study, the association between these two—antibiotic and anti‐inflammatory medication use, and ADHD—was seen as a decrease in the use of these drugs in the ADHD cohort after ADHD treatment initiation. Based on this real‐word evidence study we cannot, however, conclude reasons behind this phenomenon. Future research is needed, for example, about possible anti‐inflammatory effectiveness of ADHD drugs, like that of selective serotonin reuptake inhibitors and serotonin and noradrenaline reuptake inhibitors [32]. Since the change was not equal between ADHD patients and their controls, we can rule out the thought that the decrease would have been solely caused by COVID‐19 that in general decreased the prevalence of bacterial infections. The reasons behind increased number of bacterial infections in ADHD patients should be studied further to better understand the underlying causes.

In our Finnish cohort, 83% of incident ADHD patients initiated ADHD medication. As expected, these patients most often used methylphenidate as their first ADHD medication, aligning with previous Swedish data, where 82% of patients receiving ADHD medication used methylphenidate as their first‐line drug [9]. We analyzed different methylphenidate products separately and saw that in children, the first drug was most often the sustained‐release methylphenidate, and in adolescents and adults, the extended‐release methylphenidate. This reflects Finnish guidelines advocating for these products to reduce risk of stimulant misuse [8]. Before changing to the second drug, children used their first methylphenidate product twice as long as adolescents or adults. In every age group, most commonly the first two drugs were the sustained and the extended‐release methylphenidate. There was more variation in the ADHD medication regimen in adults, although guanfacine was used by children only, as indicated. Treatment practices for ADHD have evolved over time, with a notable expansion in the availability and reimbursement of medications during the study period. For example, initially, patients over 25 years old had to apply for reimbursement for methylphenidate, while younger patients received it automatically. This was changed in 2017 and brought broader access to medications. Lisdexamphetamine was introduced to the market and gained reimbursement status in 2014 with a broader reimbursement for adults in 2018. Dexamphetamine came to Finnish market in 2016.

In Europe, ADHD medication has been approved as monotherapy, and there are only a few studies demonstrating real‐world evidence of the medication combinations. Combinations of ADHD drugs were rare in our study, primarily involving instant‐release methylphenidate alongside the initial medication. Approximately 30% of the patients using instant‐release methylphenidate used it with some other ADHD medicine. Although the use of guanfacine and dexamphetamine was uncommon, a relatively large portion, about 30% of children using guanfacine and about 50% of adults using dexamphetamine, used these drugs together with other ADHD medications. These patterns align with Canadian approvals for such combinations [33].

Primary adherence to ADHD drugs was very high in our data, with over 80% of the patients purchasing the prescribed medicine within 10 days, which indicates that most patients are motivated to use their medication. Against expectations, most patients bought medication for 1 month only and only minority for longer time at once, even if the reimbursement system enables purchases for up to 3 months. This indicates that ADHD drugs were not massively misused, as large purchases would be indicative of it. Unlike expected, larger purchases were not seen even in the end of the calendar year, even if the reimbursement system favors that too. However, school‐age children exhibited seasonal variation with fewer purchases during summer holidays indicating drug holidays.

ADHD medication persistence was the longest in the youngest age group, and even better in boys than in girls. According to the guidelines, ADHD medication use in patients under 6 years requires extra‐careful caution [8], and ADHD medications are indicated starting from the age of 6 years in Finland. The result suggests that young patients to whom the medication has been initiated have benefited from it, and the parents, as they are responsible for the administration, have been committed to the treatment. Methodologically, it is notable that age in Figure 3a refers to age at the time of the first purchase, and most probably, patients in this age category have turned 6 years during the follow‐up. In 6–12‐year‐old children, persistence with ADHD medication was particularly high in the beginning but decreased after 1 year and during the follow‐up. If there is a clear need for ADHD medication and its outcomes are beneficial it may last for years, and withdrawing medication with good response is not meaningful. When getting older, children do not necessarily need the medication as earlier. In adolescents, the persistence was the lowest, but the time effect cannot explain the high discontinuation rate: more than half of the patients discontinued ADHD medication within 1 year. This may stem from symptom alleviation, adverse effects, or refinement of diagnoses. Despite independent responsibilities in this patient group, supporting both adolescent patients and their families not only at initiation but during the medical treatment is crucial [11]. Our data showed that 25% of patients with ADHD identified in adulthood maintained continuous ADHD medication for 5 years. This is consistent with Danish findings, although the discontinuation definition by Ishøy et al. was less strict (up to 12 months) compared to ours (6 months) [13].

When observing persistence with different drugs, we saw that the majority of the users discontinued the medication of a particular drug within 1.5 years. Lisdexamphetamine and guanfacine exhibited the longest persistence, and especially adult patients were persistent with lisdexamphetamine. About 75% of instant‐release methylphenidate and atomoxetine users discontinued the medication within 1 year, and almost 50% of instant‐release methylphenidate users and about 30% of dexamphetamine users withdrew the medication in 3 months. When analyzing all methylphenidate products together, their combined persistence was generally high (data not shown). In the recent Danish study, it was concluded that comorbid eating disorder, intellectual disability, and sleep disorder were associated with longer persistence with ADHD medication [13].

Our results show that persistent ADHD medication use consists of different drug products. Switching medications, common in ADHD management, underscores the importance of individualized treatment plans to optimize outcomes and minimize side [1, 8]. While adherence was generally high, and especially the youngest patients seemed to benefit from the medication, achieving optimal treatment may require multiple adjustments for some patients.

## 5 Conclusion

The majority of the Finnish ADHD patients (95%) purchased the ADHD medication prescribed for them and 80% bought it within 10 days, which indicates high primary adherence. The youngest children remained on continuous ADHD medication the longest. Adolescents discontinued their medication early compared to other age groups. In school‐age children and adolescents, we saw gaps in medication purchases during the summer holiday months.

ADHD treatment initiation was associated with a decrease in concomitant medication use. In children, the use of antibiotics and anti‐inflammatory drugs decreased more than in their controls of same age after ADHD treatment initiation. Incident adult ADHD patients used antidepressants remarkably prior to ADHD identification, but there was also a remarkable decrease in antidepressant use after the initiation of ADHD treatment, which is a novel finding of this study.

## Author Contributions

Conceptualization: E.W., J.J., A.P., and S.L. Data curation: A.K., and P.R. Formal analysis: A.K., and P.R. Funding acquisition: E.W., and J.J. Investigation: E.W., T.P., I.I.‐M., J.J., M.J.K., A.P., and S.L. Methodology: T.P., P.R., and M.J.K. Project administration: E.W., and I.I.‐M. Resources: E.W., and I.I.‐M. Software: A.K., and P.R. Supervision: M.J.K., A.P., and S.L. Validation: P.R., and M.J.K. Visualization: A.K. Writing – original draft: E.W., T.P., A.K., and I.I.‐M. Writing – review and editing: J.J., M.J.K., P.R., A.P., and S.L. All authors have contributed to the manuscript substantially and fully meet the criteria 1–4 of the International Committee of Medical Journal Editors (ICMJE) for authorship. They have agreed to the final submitted version.

## Ethics Statement

The study permit was granted by the Finnish Social and Health Data Permit Authority Findata with diary number THL/3461/14.02.00/2022. The study protocol was subjected to ethical consideration during the Findata permit process. No separate ethical committee process was required according to local legislation.

## Consent

According to local legislation, informed consents were not required due to the secondary nature of the register data.

## Conflicts of Interest

E.W. and J.J. are employees of Takeda Oy but have no Takeda stock or stock options. T.P., A.K., I.I.‐M., P.R., and M.J.K. are employees of Oriola, and Oriola received research funding for this study by Takeda Oy. A.P. has received consultation fees from Takeda and Biocodex, and S.L. from Takeda, Lundbeck, and Janssen.

## Peer Review

The peer review history for this article is available at https://www.webofscience.com/api/gateway/wos/peer‐review/10.1111/acps.70007.

## Supporting information


**Figure S1.** Primary adherence in time from prescription to purchase of an ADHD drug by age groups.

## Data Availability

The data that support the findings of this study are available from the Finnish Social and Health Data Permit Authority Findata. Restrictions apply to the availability of these data, which were used under license for this study. The data can be received with permission of Findata: https://findata.fi/en/permits/.
