# Supplementary figures and images for: Use of Antidepressants Decreased After Initiation of ADHD Treatment in Adults—A Finnish Nationwide Register Study Describing Use of ADHD and Non‐ADHD Medication in People With and Without ADHD
Source: Acta Psychiatr Scand. 2025 Jun 27;152(3):203–15. doi: 10.1111/acps.70007 (PMC12318640; doi:10.1111/acps.70007)

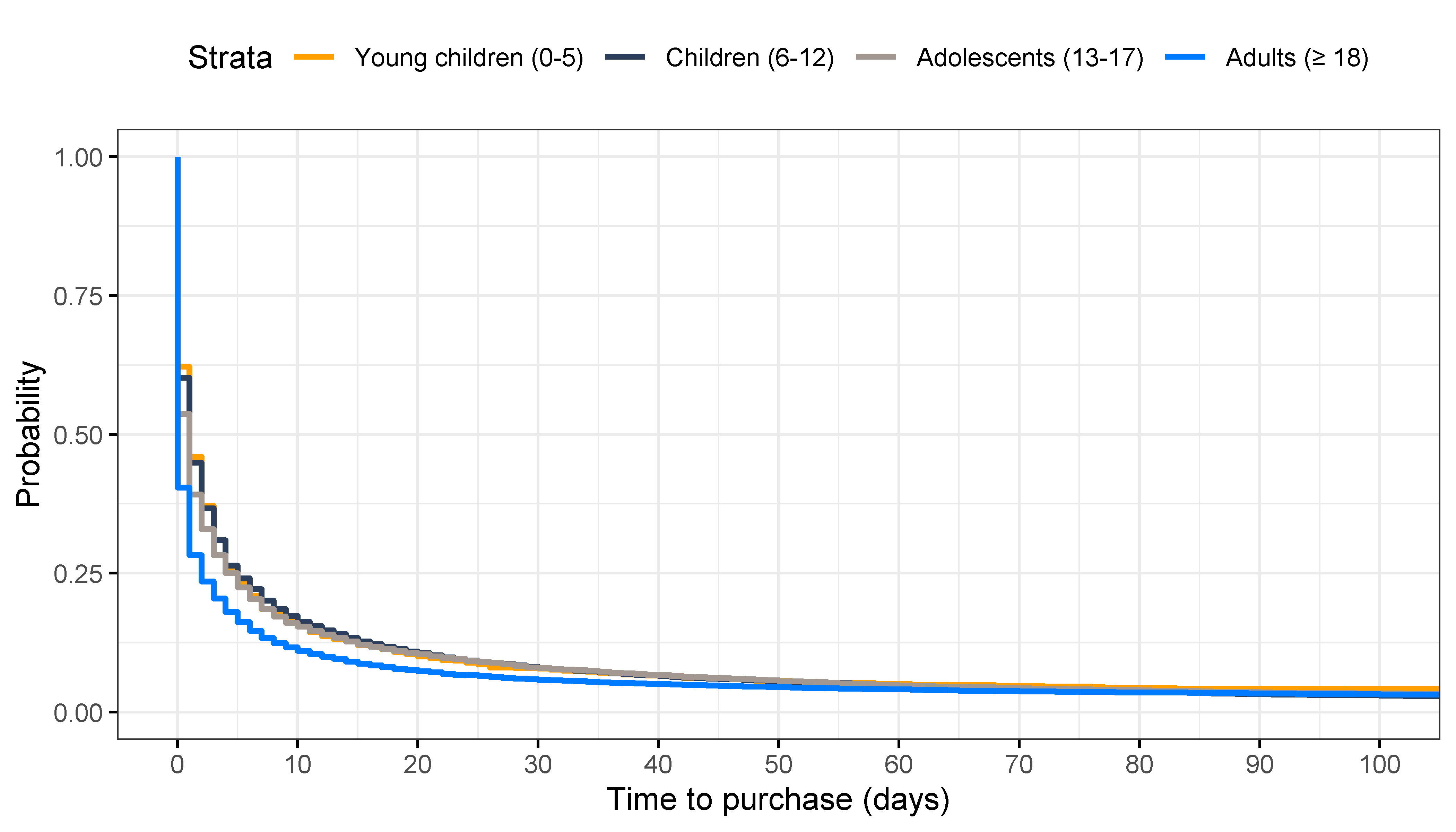

Supplement: Supplementary file 1 — Figure S1. Primary adherence in time from prescription to purchase of an ADHD drug by age groups. [file ACPS-152-203-s001.png]
